# A New Paradigm for Pediatric AML: Improving the Pipeline for Treatments Targeting Cytogenetic and Molecular Alterations

**DOI:** 10.3390/cancers18162686

**Published:** 2026-08-19

**Authors:** Camila Ayerbe, Aaron E. Fan, Ryan Scanlan, Reeja Raj, Samanta Catueno, Anwesha Ray, Huber Aguirre, David McCall, Michael Roth, Miriam B. Garcia, Cesar Nunez, Irtiza N. Sheikh, Guillermo Garcia-Manero, Branko Cuglievan, Amber Gibson

**Affiliations:** 1 Department of Pediatrics, University of Texas Southwestern, Dallas, TX 77030, USA; 2 Division of Pediatrics, The University of Texas MD Anderson Cancer Center, Houston, TX 77030, USAsscatueno@mdanderson.org (S.C.);; 3 Department of Pharmacy, The University of Texas MD Anderson Cancer Center, Houston, TX 77030, USA; rscanlan@mdanderson.org; 4 Division of Pediatrics, Leukemia/Lymphoma Section, The University of Texas MD Anderson Cancer Center, Houston, TX 77070, USA; 5 Department of Pediatric Stem Cell Transplantation and Cellular Therapy, The University of Texas MD Anderson Cancer Center, Children’s Cancer Hospital, Houston, TX 77030, USA; 6 Department of Leukemia, The University of Texas MD Anderson Cancer Center, Houston, TX 77030, USA

**Keywords:** pediatrics, leukemia, cytogenetics, clinical trials, high-risk, targeted therapies

## Abstract

Pediatric acute myeloid leukemia (AML) is a highly heterogeneous malignancy, with cytogenetic and molecular abnormalities playing a critical role in determining prognosis and guiding treatment decisions. Despite therapeutic advances, patients with high-risk genetic mutations and translocations continue to experience suboptimal outcomes. As new targeted therapies emerge, the treatment of pediatric AML could undergo a paradigm shift, where “one-size-fits-all” chemotherapy is no longer the only frontline approach. Identifying genetic markers inform risk stratification and have greater impact on shaping the therapeutic approach, including the integration of targeted therapies such as FLT3 and menin inhibitors into frontline therapy. Furthermore, pediatric AML treatment options are being driven by recent discoveries in adult AML, broadening their clinical trials to include pediatric patients, in part due to the RACE for Children Act that went into effect in August 2020. This review identifies the most prevalent high-risk cytogenetic lesions in pediatric AML, emphasizing their incidence, prognostic significance, and implications for clinical management. By synthesizing current research on these key genetic abnormalities and their associated therapies, we aim to provide an updated perspective on the evolving landscape of high-risk pediatric AML management that can then lead to the establishment of an agile framework to rapidly evaluate, approve, and deploy novel agents.

## Simple Summary

Pediatric acute myeloid leukemia (AML) is a complex disease that has traditionally been treated with chemotherapy, but some forms of AML have not responded well to this approach. Recent studies have identified a diverse array of genetic changes that show differences between adult and pediatric AML, resulting in a shift in how clinicians think about the best way to treat this disease. The goal of this review is to provide an overview of key genetic targets and clinical studies that lay the foundation for establishing a framework to bring cutting-edge treatment options to the front line for treating pediatric AML.

## 1. Introduction

Leukemia is the most common childhood malignancy, accounting for approximately 30% of all pediatric cancer diagnoses worldwide [1]. Acute myeloid leukemia (AML) represents ~20% of these diagnoses, and its complexity contributes to lower survival rates compared to its acute lymphoblastic leukemia (ALL) counterpart [2,3]. While overall survival (OS) for pediatric AML has improved to around 70%, the incidence of relapse remains high, and prognosis is dependent on high-risk cytogenetic and molecular alterations [4]. A growing emphasis on identifying and understanding these genetic aberrations and pathways has unearthed the wide genomic heterogeneity of AML, and it has also in some instances enabled coalescence of AML groups based on their disease biology, as in the case of menin (*MEIS*/*HOX*) dependent leukemias, which could harbor several divergent genetic aberrations [5,6].

Discoveries of several genomic aberrations in AML subgroups over the last two decades have enabled clinicians to understand disease ontogeny, make diagnostic algorithms more precise, promote the use of targeted therapy, and risk-stratify patients based on predicted clinical outcomes. These elements are driving a paradigm shift in pediatric AML, where a “one-size-fits-all” chemotherapy approach is no longer the only option for treatment. Rather, each aberration can be treated as a distinct genotype representative of a discrete condition with varying prognoses, necessitating the development of more personalized therapies for frontline treatment. The Research to Accelerate Cures and Equity (RACE) for Children Act has opened a pipeline for novel therapies first developed in adults to be evaluated in pediatrics at a faster pace. This has enabled enrollment of patients ≥12 years old in adult trials and expanded treatment opportunities for children with rare genotypes and limited prior therapies.

In this review, we highlight the incidence and significance of various high-risk genetic aberrations in pediatric AML. Discussing these cytogenetic changes provides a background for the discussion of pediatric AML treatment trending toward a personalized approach. Additionally, we examine their prognostic implications and potential targeted therapies, with the goal of providing a comprehensive overview of how these aberrations influence and change clinical management strategies from previous approaches.

## 2. Risk Stratification in Pediatric AML

Traditionally, AML has been stratified as favorable, intermediate, or adverse based on cytogenetic aberrations and available data on their outcomes for the respective period. With better and wider availability of genetic techniques, newer parameters have been added over the years to these stratification models in both pediatrics and adults [7]. The risk stratification system established by the European LeukemiaNet (ELN) is one of the most widely used in adult AML and is periodically updated based on available data. The latest risk stratification in 2022 uses a combination of cytogenetic aberrations and gene alterations commonly involved in AML to stratify patients into three risk strata [8]. Although pediatric AML is biologically distinct from adult AML, its risk stratification extrapolates from adult models such as the ELN [7]. An international consensus recently published recommendations based on ELN 2022 that advise on the diagnosis, monitoring, and treatment of pediatric AML [9]. To give insight, adult AML is enriched with myelodysplasia-related and *TP53* mutations but is rare in pediatric AML, which is in turn more enriched with *KMT2A* rearrangements and *RAS* pathway mutations. Additionally, the tolerability of chemotherapeutic agents and availability/approval of targeted drugs are different in the pediatric and adult populations, affecting outcomes even in genetically similar AMLs.

## 3. Common Genetic Aberrations in Pediatric AML

Compared to adults, a greater portion of pediatric AML is characterized by cytogenetic aberrations [10], ranging from normal karyotype to the more unfavorable complex karyotype, translocations, or rearrangements, of which the most relevant aberrations are summarized in Table 1. These alterations vary in prevalence and prognosis, and they represent potential therapeutic targets that will be discussed later. Collectively, these genetic aberrations guide risk stratification and targeted therapy development in the current age of precision oncology.

## 4. *KMT2A* Rearrangement (*KMT2A*-r)

Lysine methyltransferase 2A (*KMT2A*) rearrangements, also known as mixed lineage leukemia (MLL), at chromosome 11q23 are common in pediatric AML, occurring in 40% of infants (<1 year of age) and 15% of children [26,27]. *KMT2A* rearrangements (*KMT2A*-r) cause an overexpression of *HOX* cluster genes and *MEIS1*, leading to uncontrolled proliferation and ultimately leukemic formation [5]. These rearrangements can be found in both myeloid and lymphoid leukemias in infants, children, and adults and are driven by the fusion of the *KMT2A* gene with the possibility of over 80 different partners [6]. Despite the large number of possible fusion partners, nine fusions account for ~90% of all *KMT2A*-r found in pediatric acute leukemias, with the most common being *KMT2A::MLLT3* [6,28]. *KMT2A*-r pediatric AML is associated with chemotherapy resistance and high relapse rates, but they also demonstrate varying outcomes within specific fusion subgroups [29,30]. A study containing 756 patients with 11q23 or MLL-rearranged pediatric AML collected from 11 different study groups noted that t(6;11)(q27;q23), t(10;11)(p12;q23), and t(10;11)(p11.2;q23) were associated with varying 5-year event free survival (EFS) (11%, 31%, and 17%, respectively) and OS (22%, 45%, and 27%, respectively) rates [13]. Furthermore, sequencing data has indicated a relationship between *KMT2A*-r and other gene alterations; for example, patients with *KMT2A*-r who also demonstrated high *MECOM* expression were associated with a poorer prognosis [31]. The divergent outcomes even in this otherwise homogeneous genetic group of *KMT2A*-r AML, with respect to the partner gene, are relevant for adequate prognostication, as well as understanding improvement in clinical trial outcomes and real-world settings.

Menin inhibitors are a newly approved class of targeted agents that are highly effective in relapsed/refractory (R/R) *KMT2A*-r AML, among other *HOX*/MIES1-driven AML subtypes. The breakthrough phase I trial AUGMENT-101 utilizing revumenib, an oral menin inhibitor, showed safety with a 63.2% overall response rate (ORR) and a 22.8 complete response (CR) in adult and pediatric patients with R/R *KMT2A* or nucleophosmin 1 (*NPM1*) AML [32]. These results led to revumenib’s rapid approval by the Food and Drug Administration (FDA) in late 2024 for adults and children older than 1 year. Resistance to revumenib arises through *MEN1* mutations or alternative mechanisms, and studies are still underway to determine the best therapy combination with menin inhibitors for refractory disease. In all, menin inhibitors have shown such strong early results that they are now being tested in the frontline setting, marking a meaningful shift away from traditional cytotoxic chemotherapy.

In addition to menin inhibition, activation of anti-apoptotic pathways has been identified in *KMT2A*-r AML, leading to the investigation of novel drug combinations involving venetoclax, a BCL-2 inhibitor [33]. Combinations of venetoclax with hypomethylating agents and chemotherapy have greatly influenced the treatment of AML in adults, reducing the need for intensive chemotherapy. In pediatrics, these combinations are being explored in both relapsed and frontline therapy [34,35]. Additional preclinical work has identified strong synergistic effects of venetoclax when combined with the bromodomain and extra-terminal (BET) inhibitor I-BET151, the kinase inhibitor sunitinib, and thioridazine, leading to enhanced mitochondrial apoptotic activation and increased leukemic cell death in both cell lines and primary samples [33]. *DOT1L* has been shown to be a key player in *KMT2A*-fusion-driven leukemogenesis along with menin. Preclinical studies combining revumenib with the *DOT1L* inhibitor pinometostat demonstrated significant synergy, highlighting the translational potential for combination therapies and the need for precise patient stratification to optimize treatment outcomes [36].

## 5. *FLT3* Alterations

FMS-like tyrosine kinase 3 (*FLT3*) receptor is expressed in early myeloid precursors and plays an important role in cell maturation and differentiation through downstream activation of the RAS, MEK, PI3K, AKT, and STAT5 pathways. *FLT3* gene aberrations are more common in adults compared to children, with occurrence rates of 10–15% in pediatric patients, compared to approximately 25% in adults [20]. Within *FLT3*, alterations can include internal tandem duplication (ITD) or tyrosine kinase domain (TKD). *FLT3*-ITD alterations are associated with poor survival outcomes compared to patients with wildtype-*FLT3* [20]. Prognostic implications of *FLT3*-TKD remain uncertain and are not included as an adverse risk in commonly used risk stratification models. Of note, adult and pediatric risk frameworks have diverged with respect to *FLT3*-ITD. Adult patients with *FLT3* alterations were considered an adverse risk feature (if not co-mutated with *NPM1*) in the previous ELN 2017, but the recent ELN 2022 update eliminated the allelic ratio from the risk classification and assigns all *FLT3*-ITD AML to the intermediate risk due to the improvement in outcomes with *FLT3* inhibitors. No reclassification or decrease in risk stratification has occurred yet for pediatric AML with *FLT3*-ITD. ASTCT guidelines still currently recommend hematopoietic stem cell transplant (HSCT) in CR1, and no ELN equivalent recommendation has yet to be adopted by pediatric cooperative trials.

*FLT3* targeted therapies developed in adult patients have led to several FDA approvals of new options for *FLT3*-altered AML. First-generation inhibitors (midostaurin, sorafenib) are considered nonspecific agents due to their off-target effects, whereas second-generation inhibitors (gilteritinib, quizartinib, crenolanib) are more specific [37]. *FLT3* inhibitors are also classified as either type I or type II, where type I inhibitors (midostaurin, gilteritinib) have activity against both *FLT3*-ITD and *FLT3*-TKD, while type II inhibitors (quizartinib, sorafenib) only act against *FLT3*-ITD [38]. While effective as a selective inhibitor, secondary FLT3 kinase domain mutations, particularly D835 and F691L, can emerge during treatment, representing a potential mechanism of acquired therapeutic resistance [39].

Sorafenib is a first-generation multi-kinase inhibitor that has been studied extensively in newly diagnosed adult AML patients. The SORAML trial utilized sorafenib or placebo in addition to standard chemotherapy in a cohort of adult patients with newly diagnosed AML. While the sorafenib treatment group showed improved EFS, a 5-year follow-up study showed no OS benefit with additional tolerability concerns due to off-target effects [40]. Therefore, sorafenib has limited utility in current clinical practice especially with newer *FLT3* inhibitors becoming available. However, a recent report from the COG trial AAML1031 demonstrated safety and improved EFS in pediatric patients with newly diagnosed high allelic ratio *FLT3*-ITD AML receiving sorafenib during induction and maintenance [41].

Gilteritinib was approved by the FDA after showing prolonged survival and increased remission rates in adult patients with *FLT3*-TKD or *FLT3*-ITD R/R AML [42]. The recent COG study AAML1831 utilized gilteritinib in their frontline regimen for pediatric patients with *FLT3*-mutated AML, but results from this portion of the study have not yet been published. However, data from a case series is encouraging with a 62% remission rate [43].

Quizartinib is a *FLT3*-selective inhibitor that was approved in 2023 for adult patients with newly diagnosed *FLT3*-ITD AML. The QuANTUM-First trial compared quizartinib with placebo in combination with standard chemotherapy during induction, consolidation, and maintenance (up to 3 years), showing an improved OS by 16 months compared to placebo with similar hazard ratios to trials using other *FLT3* inhibitors [44]. It is important to note that quizartinib was found to have an increased risk of QTc prolongation, with two patients in the quizartinib group suffering cardiac arrest. As a result, the FDA issued a black box warning outlining the risk of QTc prolongation, torsades de pointes, and cardiac arrest. Interestingly, a recent study found that quizartinib/chemotherapy combination therapy prolonged EFS and OS compared to placebo in patients with newly diagnosed *FLT3*-ITD-negative (wildtype) AML [45]. These findings suggest that even patients with *FLT3* wildtype AML may still benefit from this therapy, challenging our understanding of the utility of *FLT3* inhibitors outside of their intended use [46]. In children, multiple trials in various phases are currently enrolling children with AML to receive quizartinib either as monotherapy or in combination with standard chemotherapy [47,48].

*FLT3* inhibitors have had a positive impact on adult survival outcomes in both *FLT3*-ITD and *FLT3*-TKD AML across induction, consolidation, and maintenance. With these improvements in outcomes, the use of *FLT3* inhibitors represents a promising frontline approach for children with a previously poor prognosis.

## 6. *NUP98* Fusions

Nucleoporin 98kD (*NUP98*) gene fusions are considered rare (overall prevalence 7.2%) and are associated with poor outcomes [14]. *NUP98* fuses with over 20 different partner genes resulting in a high expression of oncogenic *HOX* cluster genes [49]. Mechanistic studies have shown that *NUP98* fusions interact with *KMT2A* to promote *HOX* gene activation. Because *KMT2A* inhibition attenuates *HOX* expression and downstream leukemic gene programs, this data supports a shared *HOX*-driven dependency in both *KMT2A*-r and *NUP98*-r AML, providing a rationale for the use of menin inhibitors in these subtypes [50].

The fusion of *NUP98* with nuclear receptor binding SET-domain protein 1 (*NSD1*) commonly occurs with *FLT3*-ITD in up to 15% of patients [51]. Patients with both *FLT3*-ITD and *NUP98::NSD1* had lower 3-year EFS and OS than *FLT3*-ITD alone (14% vs. 33%, 31% vs. 48%, respectively). This should be explored as an opportunity to co-target *NUP98*-r and *FLT3*-ITD in these patients with poor outcomes. Among *NUP98* target genes, the loss of CDK6 plays a critical role in *NUP98* fusion-driven leukemogenesis, a characteristic also observed in other AML subtypes, including *KMT2A*-r and *RUNX1::RUNX1T1*-core-binding factor (CBF) AML. The CDK4/6 inhibitor palbociclib, approved for breast cancer treatment, has shown strong anti-proliferative effects and promoted differentiation in murine AML models with *NUP98::KDM5A*, *NUP98::DDX10*, and *NUP98::NSD1* fusions, as well as in human *NUP98*-r leukemic cells, but these treatments still remain under early investigation in pediatric patients [52].

## 7. *UBTF*

Upstream binding transcription factor (*UBTF*) plays a crucial role in ribosomal RNA transcription. In patients with *UBTF*-ITD, *FLT3* or *WT1* alterations frequently co-occur. The overall incidence is low (~4% of pediatric cases), and is associated with poor outcomes [53]. A study conducted by the Japanese Leukemia/Lymphoma Study Group (JPLSG) found that high PR domain-containing 1 (PRDM16) expression or trisomy 8 also contributed to poor prognosis [21]. The pathogenesis of *UBTF*-ITD is still being investigated, but it has been suggested that *KMT2A* activity is present, resulting in dysregulated *HOX* gene clusters. *UBTF*-ITD acts as a gain-of-function alteration, mislocalizing to dysregulated loci, including *HOXA/HOXB* and *MEIS1*, while co-occupying sites with *KMT2A* and menin. Sustained *UBTF*-ITD chromatin localization drives stemness, proliferation, and transcriptional dysregulation [22]. This mechanism supports the use of menin inhibitors, which have shown anti-leukemic activity in *UBTF*-ITD AML. In preclinical studies, revumenib reduced tumor growth, promoted cellular differentiation, and normalized leukemic signatures, offering a promising therapeutic approach for high-risk *UBTF*-ITD AML [22].

## 8. *CBFA2T3::GLIS2*

The *CBFA2T3::GLIS2* gene fusion is characteristic of the “RAM phenotype” and is caused by the inversion of chromosome 16. In this fusion, *GATA1* transcription factors are downregulated, preventing megakaryocytic cell differentiation [54]. *CBFA2T3::GLIS2* is typically seen in non-Down syndrome acute megakaryoblastic leukemia (non-DS-AMKL), which accounts for about 8–10% of childhood AML cases and portends a poor prognosis [55]. *GLIS2* is closely related to the GLI family of transcription factors that function along the hedgehog pathway regulating stem cell differentiation. In preclinical studies, overexpression of GLI1 promoted tumor growth, whereas GLI1 antagonism resulted in leukemia apoptosis and cell arrest, representing a potential mechanistic target for therapy [56].

*FOLR1*, which encodes folate receptor α (FOLR1), has been identified as a specific and promising therapeutic target in pediatric *CBF*/*GLIS*-positive AML [57]. Preclinical evaluations using STRO-002, a FOLR1-directed antibody–drug conjugate, demonstrated potent, target-dependent cytotoxicity against FOLR1-expressing leukemic cells while sparing normal hematopoietic cells; this drug was tested for CBFA2T3::GLIS2 AML in NCT06679582, but the trial was terminated early and results have not yet been published. Preclinical evaluation of FOLR1-directed chimeric antigen receptor T-cells (CAR-T) showed efficacy against *CBFA2T3::GLIS2* AML in vitro and in xenograft models [58], and this therapy is now being explored in NCT06609928. Novel immunotherapeutic approaches for treating this aggressive pediatric leukemia are desperately needed, and the potential benefit of targeting FOLR1 highlights the utility of repurposing therapies initially developed for solid tumors for AML when appropriate.

## 9. KAT6A Fusions

KAT6A is a lysine acetyltransferase, previously referred to as monocytic leukemia zinc finger protein (MOZ or MYST3), which can fuse to histone acetyltransferases CREB binding protein (CREBBP or CBP) or E1A binding protein (EP300) [28]. KAT6A functions to maintain proper hematopoietic cell function, and when deleted, may lead to depletion of hematopoietic stem cells [59]. KAT6A fusions in pediatric patients are rare with limited prognostic and clinical implications, but it is strongly associated with 25% of congenital AML [4]. Recent preclinical data suggests that targeted inhibition of KAT6A or KAT7 using PF9363, a commercially available MOZ inhibitor, can potentiate the efficacy of menin inhibitors in treating menin-resistant MLL AML and *NUP98*-r AML, providing further evidence of protein interactions with other pediatric AML genetic abnormalities [60]. WM-1119 is another MYST acetyltransferase inhibitor that was shown to block the proliferation and promote differentiation of leukemic myeloid cells [61]. Although these data are still in preclinical phases, combination therapies with KAT6A inhibitors may represent an alternative therapy for this treatment-resistant AML.

## 10. *MNX1* Rearrangement

The translocation of (7;12)(q36;p13) results in the relocation of the motor neuron and pancreas homeobox 1 (*MNX1*) on chromosome 7 within the *ETV6* locus on chromosome 12. This is most commonly seen in infant patients with a median age of 6 months, and the incidence is 18–30% [62]. Currently, there are no targeted therapy strategies for this aberration, and survival outcomes are usually poor [17]. However, preclinical data suggests that combination therapies including DNA methyltransferase inhibitors may contribute to *MNX1* suppression, leading to leukemic cell death [63].

## 11. MECOM 3q26.2

The *MDS1* and *EVI1* complex locus (MECOM) is located on chromosome 3q26.2 locus. In the typical or classic rearrangement, MECOM is juxtaposed to regulatory elements such as *GATA2* or other enhancers, leading to aberrant MECOM overexpression. The most common additional aberration is monosomy 7 or 7q deletion, reported as high as 39% of patients [18]. This results in blocked myeloid differentiation, enhanced self-renewal, uncontrolled proliferation, and widespread epigenetic reprogramming. Atypical MECOM rearrangements also involve 3q26 and have different cytogenetic patterns, but they are less clearly defined. However, studies have demonstrated that these rearrangements functionally overexpress EVI1 and have altered *GATA2* expression, effectively concluding that atypical MECOM should be classified together with classic MECOM rearrangements [64].

Both classic and atypical MECOM rearrangements are often cryptic; thus, the incidence has ranged from 1 to 28% in cytogenetic reviews of pediatric AML patients [18,65,66]. Patients with MECOM overexpression frequently demonstrate chemotherapy resistance, correlating with high rates of treatment failure, inferior outcomes, and limited therapeutic progress. Despite ongoing research, clinical outcomes remain poor and no targeted therapy is approved. BET inhibitors, such as mivebresib, are being explored in preclinical contexts, combining with inhibitors of apoptosis (IAP) proteins, such as LCL161, and they show in vivo rationale for exploration in this population [67]. Ongoing early-phase studies are testing alternative strategies, including transcriptional CDK inhibition (NCT04588922), and Poly(ADP-ribose) polymerase 1 (PARP1) inhibition. There is an urgent need to identify novel agents for pediatric patients with MECOM-r AML.

## 12. *DEK::NUP214*

The *DEK::NUP214* fusion has a rare overall incidence (1–2% in childhood AML), and it is classified as a poor risk category in both pediatric and adult AML [68,69]. Patients presenting with *DEK::NUP214* fusion tend to have late-onset diagnosis with a median age of ~10 years and are mostly male. These leukemias can also have a moderately high incidence of *FLT3*-ITD, making them candidates for *FLT3* inhibitor-based therapy. Although pediatric patients historically had poor outcomes with 5-year EFS and OS rates around 14–18%, intensified therapies such as HSCT and *FLT3* inhibitors significantly improved survival, with recent trials (AAML1031) showing 5-year EFS and OS rates of 71% and 94%. The use of HSCT in CR1, especially for those with *FLT3*-ITD mutations, was a critical factor in producing these improved outcomes, demonstrating significant increases in EFS compared to patients in CR1 who received traditional chemotherapy alone [19].

## 13. 7q Abnormalities

Monosomy 7 (−7) is a common abnormality found in pediatric myelodysplastic syndrome (MDS) but is less frequent in de novo pediatric AML. It is frequently observed in the context of germline predisposition syndromes (e.g., *GATA2* deficiency, SAMD9/SAMD9L syndromes, and inherited bone marrow failure syndromes), underscoring the importance of an initial genetic evaluation. Deletion of the long arm of chromosome 7 [del(7q)] is also rare, occurring in only 4–5% of pediatric AML cases [23]. In a large international retrospective study of children and adolescents with AML, −7 was associated with a 5-year OS and EFS of 35% and 28%, respectively; del(7q) on the other hand had a 5-year OS and EFS of 43% and 39%, respectively [23].

## 14. Signaling Pathway Mutations

The RAS gene family is made up of HRAS, KRAS, and NRAS, which function to regulate cell division through downstream signal transduction pathways [24]. There are FDA-approved agents that target the RAS pathway for the management of other adult malignancies along with additional inhibition of downstream targets MAPK and PI3K. RAS inhibition in pediatric malignancies has not yet been studied in clinical trials but could serve as a potential targeted therapy option given RAS involvement in various pediatric hematologic malignancies [70].

KIT is a tyrosine kinase receptor that is expressed on hematopoietic cells and plays a role in the process of hematopoiesis [71]. Mutations in KIT are found in only 4–5% of pediatric AML patients but are quite prevalent in those with CBF-AML comprising approximately 30% of patients. The prognostic implications of KIT mutations in CBF-AML are controversial with studies showing mixed results; in adults, CBF-AML patients have a favorable risk stratification regardless of KIT status [72]. However, one meta-analysis suggests that they are associated with a poor prognosis [71].

## 15. Mutation of Epigenetic Regulators

Epigenetic regulators control gene expression through DNA methylation and histone modification [73]. In pediatric AML, recurrent alterations are observed in genes including Ten-Eleven Translocation 2 (TET2), Isocitrate Dehydrogenase 1/2 (IDH1/2), Enhancer of Zeste Homolog 2 (EZH2), DNA (cytosine-5)-Methyltransferase 3 Alpha (DNMT3A) and Additional Sex Combs Like-1 (ASXL1). These mutated genes are frequently reported in adult patients, but in pediatric patients, the incidence may be as low as 1–4% [74]. Importantly, there are FDA-approved epigenetic therapies aimed to reverse this dysregulation. These agents fall broadly in the categories of hypomethylating agents (HMAs) and histone-modification agents. HMAs such as azacitidine and decitabine in combination with venetoclax have become the backbone therapy for many adult AML patients. In pediatrics, although the mutation rates are far lower, studies have demonstrated clinical benefits. Clinical trials using HMAs alone or in combination with chemotherapy have spanned the past decade, with other combinations using novel agents (e.g., venetoclax, menin inhibitors) currently also being explored (NCT06191978) [75]. HMA as epigenetic primers before chemotherapy is a new area of interest in both adult and pediatric AML. The AML16 trial assessed epigenetic priming with decitabine or azacitidine in children with newly diagnosed AML (NCT03164057), showing that decitabine was superior to azacitadine, correlating with lower total genome-wide methylation burden and increased OS [76]. Histone-modifying agents include histone deacetylase (HDAC) inhibitors such as vorinostat, BET inhibitors, and associated histone methyltransferase inhibitors including DOT1L inhibitors (pinometostat), EZH2 inhibitors, and LSD1 inhibitors. IDH inhibitors also represent a class of drugs that may have a therapeutic effect in pediatric AML despite low frequencies of occurrence.

## 16. Emerging Treatment Strategies for Pediatric AML

Current treatments for pediatric AML include combinations of intensive chemotherapy (cytarabine, anthracyclines, and/or etoposide), targeted therapies where relevant, epigenetic priming, consolidation with HSCT, and maintenance therapy post-HSCT in patients with intermediate/adverse risk AML. The recent COG AAML1831 phase 3 trial compared conventional chemotherapy to the use of CPX-351, a novel liposomal formulation combining cytarabine and daunorubicin, combined with gemtuzumab ozogamicin, an anti-CD33 monoclonal antibody. However, this study was terminated early due to no observed benefit while sustaining higher rates of toxicity [77]. The St. Jude AML97 trial demonstrated the synergistic effect of cytarabine with cladribine, a purine analog, leading to 5-year EFS and OS of 48.7% and 53.8%, respectively, for de novo AML patients, improving to 57% and 62% after treatment adjustments [78]. Other combination regimens include FLAG (fludarabine, cytarabine, granulocyte colony-stimulating factor) with or without idarubicin (IDA), and FLAG-IDA + venetoclax, which have been studied in adult patients with newly diagnosed or R/R AML. Results showed complete remission in 89% of patients, with a higher number being able to proceed to HSCT [79,80]. In recent studies for pediatric AML, FLAG-IDA +/−venetoclax as frontline therapy was shown to be safe, with many patients achieving remission and proceeding to HSCT, representing a potential shift in the initial treatment approach [35,80,81].

In the United States, there are currently 30+ clinical trials enrolling pediatric patients, utilizing single-agent and combination therapies targeting disease-specific aberrations (summarized in Table 2). The success of menin inhibitors has prompted a flurry of development to expand their use to frontline regimens, as well as new combination therapies. Bleximenib, enzomenib, and ziftomenib are other classes of menin inhibitors that are currently being investigated, of which preliminary results demonstrate acceptable safety profiles with ongoing dose optimization [82,83]. Some of these trials have now extended their eligibility criteria to include pediatric populations.

In the relapsed setting, the combination of venetoclax with menin inhibitors aims to target multiple pathways at once to potently block leukemic proliferation. A phase I/II study utilized revumenib combined with the hypomethylating agent decitabine and venetoclax in adult and pediatric (age ≥ 12) patients with *KMT2A*-r, *NPM1*, or *NUP98*-r relapsed AML. Their preliminary results demonstrated safety, with no treatment-related early mortality and manageable adverse events. Furthermore, the patient cohort had high rates of remission [84]. Ziftomenib in combination with venetoclax/azacitadine is currently being investigated in a phase I trial for adults with *KMT2A*-r and *NPM1* relapsed AML. An interim analysis showed tolerability with no dose-limiting toxicities and preliminary efficacy, with pediatric patients currently enrolling [85].

Targeted AML treatment requires close attention to mechanism-specific toxicities coupled with proactive monitoring. QTc prolongation associated with quizartinib is typically manageable with serial electrocardiographic monitoring, correction of electrolyte abnormalities, avoidance of concomitant QT-prolonging agents and strong CYP3A4 inhibitors, and dose modification or interruption when needed [86,87]. Menin inhibitors can induce differentiation syndrome (DS), an inflammatory complication of leukemic maturation characterized by fever, weight gain, edema, leukocytosis, hypoxia, pleural or pericardial effusions, and end-organ dysfunction. Early recognition of DS, prompt initiation of corticosteroids, cytoreduction for leukocytosis when indicated, and temporary treatment interruption in severe cases are critical to minimizing morbidity while preserving the therapeutic benefit of differentiation-based treatment [88,89]. Cytopenias and febrile neutropenia remain common across these targeted approaches and require standard monitoring and supportive care.

## 17. Expanding the Pipeline of Adult Therapies into Pediatric AML

Investigation of novel approaches to treating AML has typically originated in adult populations. In recent years, adult clinical trials have taken a precision medicine approach, attacking specific targets that characterize the patient’s leukemia, with plans to eventually expand access to children (Table 3). Such therapies include new classes of drugs targeting CD123, which is highly expressed in some AML and MDS. APVO436 and AZD9829 are both bispecific T-cell engagers that have shown preclinical efficacy and are currently being investigated in clinical trials [90,91,92]. CER-1236 is a therapy that leverages the body’s immune system by using an autologous chimeric engulfment receptor T-cell that mediates the targeted phagocytosis of AML cells [93]. Other therapies inhibit relevant metabolic pathways vital to leukemic cell survival. Tambiciclib (SLS009) inhibits CDK9, which is a kinase important in transcription of oncogenes of several malignancies. In AML, tambiciclib is being investigated in a phase 2 trial combining it with azacitadine and venetoclax [94]. Multi-kinase inhibitors such as adrixetinib (Q702) and tuspetinib (HM43239) are oral agents that are being tested alone or in combination with other therapies [95,96]. Many of these therapies are first-in-human and have received FDA fast-track designation, which is critical for the rapid development and assessment of these new treatments. Although there are differences between adult and pediatric leukemias, when it comes to treatment, there are shared targets and pathways that can be exploited. As new treatments become available, it will be important to regularly assess the expansion of these adult trials to include pediatric populations. The RACE for Children Act provides requirements for trial sponsors to submit a pediatric study plan, or justify deferral or waiver for any new molecularly targeted agent whose target is relevant to the growth or progression of a childhood cancer. This approach is satisfiable in the relapsed population, and single-arm studies may be satisfactory to regulators, but the regulatory criteria for pediatric cohorts to move from the relapsed setting to frontline trials are difficult to overcome. Regulators permit single-arm studies on a case-by-case basis, but only under narrowly specified conditions, such as when randomization would be unethical, the natural history is well characterized and unlikely to improve without intervention, or the anticipated treatment effect is expected to be substantially greater than the predictable course [97,98]. These criteria are difficult to satisfy when pediatric AML is viewed as a single entity without regard to is molecular drivers or when relapse and frontline populations are viewed as exchangeable. Thus, assumptions about the expected disease course become less reliable, supporting the use of external controls over the single-arm design.

**Figure 1:**
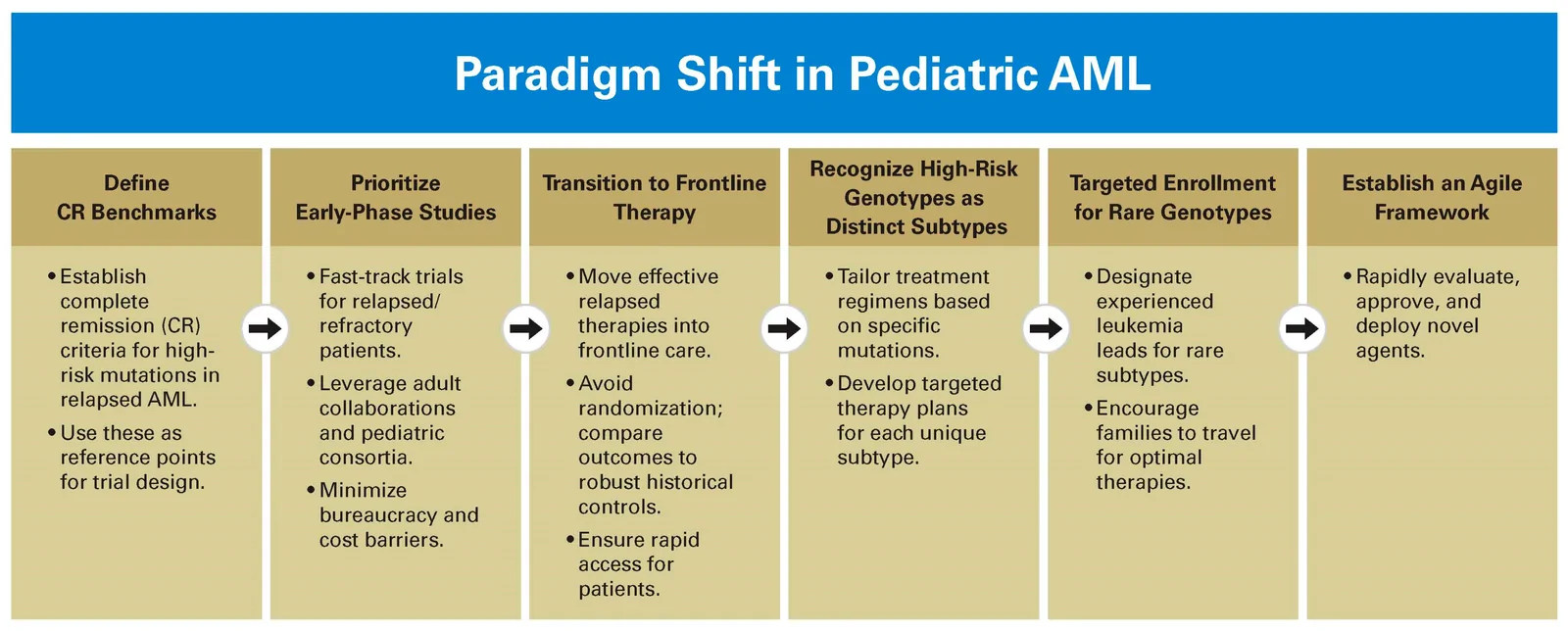
Steps to generating a paradigm shift in pediatric AML.

To improve outcomes in pediatric high-risk AML, a paradigm shift is urgently needed (Figure 1). Defining complete remission benchmarks for these mutations in the relapsed setting is key to informing trial design without unnecessary delays. High-risk genotypes should also be recognized as biologically distinct AML subtypes, each warranting tailored, target-specific regimens. Prioritizing early-phase studies encourages focused efforts at select, specialized centers with clear designation of leukemia leads experienced in managing these subtypes, addressing the issues of study heterogeneity. Furthermore, this allows the ability to leverage collaborations with adult programs or streamlined pediatric consortia that bypass the bureaucratic hurdles and cost escalations typical of large cooperative group trials. Importantly, therapies demonstrating safety and efficacy in the relapsed setting should then be swiftly transitioned to frontline therapy, ideally without randomization, by comparing outcomes to robust historical controls, or by embedding novel agents within randomized or linked designs. This approach requires the cooperation of relevant stakeholders, such as institutions and pharmaceutical companies, enabling timely access to potentially curative treatments. As such, families who are willing to travel for the most promising therapies should be supported, regardless of socioeconomic background, and efforts should focus on rapidly enrolling the appropriate patients to maximize trial efficiency and accrual. Altogether, these efforts can facilitate breakthroughs in targeted therapy, translating into cures for children with minimal delays.

The evidence supporting pediatric AML treatment approaches remains heterogeneous, with many studies limited by small sample sizes, non-randomized retrospective designs, and differences in risk stratification and outcome reporting across institutions and groups. Furthermore, clinical and methodological heterogeneity across patient populations, molecular subgroups, treatment protocols, and response definitions can also contribute to variability. Consistent with recent AML consensus statements, these limitations underscore the need for standardized risk classification and outcome reporting to facilitate more robust synthesis and comparisons across studies, which can then drive this paradigm shift further.

## 18. Conclusions

Pediatric AML remains a biologically and clinically heterogeneous disease, and while strides have been made in identifying key genetic drivers, translating these discoveries into effective, accessible therapies for children has been delayed in comparison to adult AML advancements. The integration of molecular diagnostics into risk stratification has refined prognostic categories and highlighted subgroups with unique therapeutic vulnerabilities, yet many high-risk genotypes continue to be associated with poor outcomes under current treatment protocols. The identification of these molecular subgroups, combined with the availability of newer therapeutic agents, has altered the risk profile of some genetic subtypes of AML. In the future, these could be adopted to further refine current risk stratification models [110]. Altogether, this underscores the critical need for innovative trial designs, focused collaboration, and targeted therapeutic strategies that can address the specific biology of these subtypes. Building sustainable frameworks to rapidly evaluate and deploy novel agents, coupled with a willingness to adapt trial structures for pediatric populations, will be essential to overcome these challenges. The future of pediatric AML treatment lies in a precision medicine approach that moves beyond one-size-fits-all strategies, tailoring therapy to the molecular profile of the disease, and ultimately improving long-term survival for children facing this aggressive malignancy.

## Figures and Tables

**Table 1:** Common mutations associated with high-risk AML. EFS, event-free survival; OS, overall survival. Survival endpoint is 5-year duration unless otherwise specified.

Genetic Aberration	Cytogenetic Change	Prevalence in Childhood AML	EFS	OS	References
KMT2A-r	Overall:	15–20%	44%	56%	[11]
	AF9(MLLT3) t(9;11)(p22;q23)	6–9%	57%	71%	[12]
	MLLT11 t(1;11)(q21;q23)	0.5–0.6%	92%	100%	[4,13]
	AFDN t(6;11)(q27;q23)	1–2%	13%	25%	[12]
	MLLT10 (10;11)(p12;q23)	3–5%	31%	45%	[4,13]
	ABI1 t(10;11)(p11.2;q23)	0.2–0.3%	17%	27%	[4,13]
NUP98	Overall:	7.2%	17%	35%	[14,15]
	NSD-1	4.8%	17%	36%	
	KDM5A	1.4%	25%	30%	
KAT6A	t(8;16)(p11;p13), t(8;22)(p11;q13)	<1%	57%	59%	[4]
CBFA2T3::GLIS2	inv(16)(p13.3q24.3)	2–3%	8%	14%	[16]
MNX1	t(7;12)(q36;p13)	4%	24% (3-year)	42% (3-year)	[17]
MECOM 3q26.2	inv(3)(q21;q26.2), t(3;3)(q21;q26.2)	2%	37% (3-year)	39% (3-year)	[4,18]
DEK::NUP214	t(6;9)(p22;q34)	1–2%	71%	94%	[19]
FLT3	ITD, TKD	5–15%	29%	32%	[20]
UBTF-ITD	Trisomy 8	4%	20% (3-year)	40% (3-year)	[21,22]
7q	Monosomy 7	2%	28%	35%	[23]
	del(7q)		39%	43%	
RAS	HRAS (11p15.5), KRAS(12p12.1), NRAS (1p13.2)	10–20%	38%	47%	[24]
KIT	c-KIT	4–5%	46% (3-year)	81% (3-year)	[25]

**Table 2:** Summary of relevant adult trials with potential to enroll pediatric AML patients.

Trial Name	NCT	Phase	Medications/Therapy/ Interventions	Route of Administration	Population (Newly Diagnosed/Relapsed/Both)	Targeted Genetic Aberration
APVO436 Phase 1b/2 Study in Patients With Newly Diagnosed AML	NCT06634394	Phase Ib/II	Drug: APVO436 Drug: Venetoclax Drug: Azacitidine	Intravenous	Newly diagnosed, CD123+ AML	CD123 bispecific T-cell engager (BiTE)
Study of AZD9829 in CD123+ Hematological Malignancies	NCT06179511	Phase I/II	Drug: AZD9829	Intravenous	Relapsed/refractory CD123+ AML	CD123 antibody–drug conjugate
Phase I Study of Q702 With Azacitidine and Venetoclax for Relapsed or Refractory Acute Myeloid Leukemia	NCT06445907	Phase I	Drug: Azacitidine Drug: Venetoclax Drug: Q702	Oral	Relapsed/refractory AML	Q702 (a receptor tyrosine kinase inhibitor), no specific genetic aberration-listed, triple kinase inhibitor of AXL, MER, CSF1R
Phase I Study of HC-7366 for Acute Myeloid Leukemia	NCT06285890	Phase I	Drug: HC-7366	Oral	Relapsed/refractory AML	Activator of GCN2, ISR kinase
CER-1236 in Patients With Acute Myeloid Leukemia (AML) (CertainT-1)	NCT06834282	Phase I	Drug: CER-1236 Drug: Cyclophosphamide Drug: Fludarabine Drug: Mesna	Intravenous	Relapsed/refractory AML (MRD positive) or TP53-mutated disease	Auto CER T-cell to enhance TIM-r phagocytosis and T-cell cytotoxicity
Clinical Trial to Evaluate the Safety, Tolerability, Pharmacokinetics and Pharmacodynamics of Tuspetinib (HM43239) in Patients With Relapsed or Refractory Acute Myeloid Leukemia (TUSCANY)	NCT03850574	Phase I/II	Drug: Tuspetinib Drug: Venetoclax Oral Tablet Drug: Azacitidine for Intravenous Infusion	Oral	Relapsed/refractory AML	Multikinase inhibitor
A Phase 1 Study of BMF-500 in Adults With Acute Leukemia	NCT05918692	Phase I	Drug: BMF-500	Oral	Relapsed/refractory AML	Oral covalent FLT3 inhibitor
A Phase I/II Study of Gilteritinib and Momelotinib for Patients With Relapsed or Refractory FLT3-Mutated Acute Myeloid Leukemia	NCT06235801	Phase I/II	Drug: Gilteritinib Drug: Momelotinib	Oral	Relapsed/refractory FLT3-mutated AML	FLT3 and JAK1/2 inhibitor
A Study of GLB-001 in Patients With Relapsed or Refractory Acute Myeloid Leukemia or Relapsed or Refractory Higher Risk Myelodysplastic Syndromes	NCT06146257	Phase I	Drug: GLB-001	Oral	Relapsed/refractory AML	Oral casein kinase 1alpha (CK1a) molecular glue degrader
Eganelisib as Monotherapy and in Combination With Cytarabine in Relapsed/Refractory AML	NCT06533761	Phase I	Drug: Eganelisib Drug: Eganelisib in combination with cytarabine	Oral	Relapsed/refractory AML	Selective PI3K-γ (phosphoinositide-3-kinase gamma) inhibitor
Open-label study of FT-2102 with or without azacitadine or cytarabine in patients with AML or MDS with an IDH1 mutation	NCT02719574	Phase I/II	Drug: Olutasidenib (FT-2102) Drug: Azacitadine Drug: Cytarabine	Oral	Relapsed/refractory AML with IDH1 mutation	Small molecule inhibitor of mutant IDH1

**Table 3:** List of currently enrolling clinical trials for pediatric patients with AML.

Drug Type	NCT	Phase	Therapy	Diagnosis	Age	Target Genetic Aberration	Available References
**Menin inhibitors**	NCT07211958	3	Revumenib + Intensive Chemotherapy	Newly diagnosed AML	≥12 years	NPM1	-
	NCT04811560	1/2	Bleximenib	Ph 1: R/R acute leukemia Ph 2: Initial diagnosis of AML and R/R disease	≥2 years	Ph 1: KMT2A, NPM1, NUP98, NUP214 alterations Ph 2: KMT2Ar, NPM1 mutation	[99]
	NCT06448013	1	Ziftomenib with Venetoclax and GO	R/R AML or MPAL (myeloid phenotype)	3–21 years	KMT2Ar, NUP98r, NPM1c, UBTF-ITD or other *HOX* pathway mutation	-
	NCT05453903	1	Bleximenib With AML-Directed Therapies	Newly diagnosed and R/R AML	≥12 years	KMT2A, NPM1, NUP98, or NUP214 alterations	[100]
	NCT05360160	1/2	Revumenib with Decitabine/Cedazuridine and Venetoclax	Newly diagnosed or R/R AML or MPAL (myeloid phenotype)	≥12 years	KMT2Ar, NUP98r, or NPM1c	[101]
	NCT06284486	1/2	Venetoclax and Revumenib	AML in first or second remission by morphology with MRD ≥ 0.1%	≥12 years	NPM1m, KMT2Ar, or NUP98r	-
	NCT06575296	1	Revumenib	Acute leukemia after allogeneic HSCT	≥2 years	NPM1m, KMT2Ar	-
	NCT04065399	1/2	Revumenib	R/R acute leukemia	≥30 days	KMT2Ar, NUP98r, or NPM1 mutation	-
	NCT06177067	1	Revumenib, Azacitidine, and Venetoclax	R/R AML or ALAL	1–30 years	KMT2Ar, NUP98r, NPM1 mutation or fusion, PICALM::MLLT10, DEK::NUP214, UBTF-TD, KAT6Ar, or SET::NUP214	-
**FLT3/kinase targeted**	NCT04293562	3	Standard Chemotherapy vs. Therapy with CPX-351 and/or Gilteritinib	Newly diagnosed de novo AML (with or without FLT3 Mutations)	≤21 years	FLT3 Mutations	[102]
	NCT04872478	1	MRX-2843	R/R AML, ALL, or MPAL	≥12 years	MerTK, FLT3	-
	NCT06262438	2	Quizartinib and Chemotherapy	Newly diagnosed AML	1 month–18 years	FLT3-ITD+ and NPM1 wildtype	-
	NCT02390752	1	TURALIO(R) (Pexidartinib, PLX3397)	R/R AML, ALL or solid tumors	3–35 years	tyrosine kinases: Kit, CSF1R, Fms and oncogenic FLT3	[103]
	NCT03934372	1/2	Ponatinib	R/R leukemias, lymphomas or solid tumors	1–17 years	BCR::ABL1	-
**BCL-2/apoptosis pathway**	NCT06191978	1	Cedazuridine/Decitabine with Venetoclax	R/R AML	2–18 years	BCL-2	-
	NCT05183035	3	Fludarabine/Cytarabine/GO with or without Venetoclax	Relapsed AML (without FLT3/ITD mutation)	29 days–21 years	BCL-2	[104]
	NCT04771572	1	Oral Administration of LP-118	R/R CLL, MDS, MDS/MPN, AML, CMML-2, MPN-BP, ALL, MF, NHL, RT, MM or T-PLL	≥13 years	BCL-2, BCL-XL	-
**CAR-T/NK engineered cells**	NCT07036250	1,2	U32 CAR-T	R/R CD38+ or CLL-1+ AML	2–65 years		-
	NCT06197672	1	CD4 CAR-T	R/R CD4+ AML	≥12 years		-
	NCT03896854	1/2	CD19 CAR-T	R/R CD19+ AML	6–65 years		[105]
	NCT05105152	1	SC-DARIC33 CAR-T	R/R CD33+ AML	≤30 years		-
	NCT06786533	1	Anti-FLT3 CAR-T	R/R AML	≥12 years	FLT3	-
	NCT02727803	2	NK Cell therapy after Chemotherapy and Umbilical Cord Blood Transplant	AML, MDS, ALL, CML, NHL, CLL, HD or MM	15–80 years		-
**Others**							
**Antibody–drug conjugate**	NCT02724163	3	Induction: Mitoxantrone and Ara-C vs. Liposomal Daunorubicin and Ara-C, GO + Induction Therapy, Consolidation: HD Ara-C vs. FLA, HSCT Conditioning: Conventional MAC (busulfan/cyclophosphamide) vs. RIC (fludarabine/busulfan)	AML/high-risk MDS/isolated myeloid sarcoma	<18 years		[106]
	NCT06994676	1	CBX-250	R/R AML, high-risk MDS and CMML	≥12 years		-
**Telomerase inhibitor**	NCT06247787	1	Imetelstat with Fludarabine and Cytarabine	R/R AML, MDS, JMML	1–18 years		-
**CDK9 Inhibitor**	NCT04588922	1/2	SLS009	R/R AML, CLL and lymphoma	≥12 years	ASXL1, BCOR, EZH2, SF3B1, SRSF2, STAG2, U2AF1 and ZRSR2 mutations	-
**IDH-2 inhibitor**	NCT03383575	2	Azacitidine and Enasidenib	IDH2-mutant MDS, AML and CMML	≥12 years	IDH2 gene mutation (IDH2-R140 or R172)	[107]
**HSCT related**	NCT03467386	1	TMLI (transplant conditioning regimen) with Post-Transplant High-Dose Cyclophosphamide	AML in first or second CR	16–60 years		-
	NCT05322850	1/2	Engineered Donor Graft (Orca Q)	HSCT Recipients in AML, ALL, MPAL or undifferentiated leukemia, CML in accelerated phase	≤50 years		-
	NCT03802695	1	Orca-Q	AML, ALL, MPAL, or high/very high-risk MDS and HSCT Recipients	12–78 years		[108]
	NCT03494569	1	TMLI with Fludarabine and Melphalan before Donor HSCT	High-risk AML, ALL or MDS	≥12 years		[109]
	NCT04262843	2	TMLI before HSCT	MDS, acute leukemia	12–60 years		-
	NCT04195633	2	Donor HSCT with Treosulfan, Fludarabine, and TBI	AML, ALL, MPAL, CML, CMML, MDS, lymphoma	≥6 months		-

ALAL, acute leukemia of ambiguous lineage; ALL, acute lymphoblastic leukemia; AML, acute myeloid leukemia; ASXL1, additional sex combs-like 1; BCL-2, B-cell lymphoma 2; BP, blast phase; CAR-T, chimeric antigen receptor T-cell; CD, cluster of differentiation; CLL, chronic lymphocytic leukemia; CML, chronic myeloid leukemia; CMML, chronic myelomonocytic leukemia; CR, complete remission; CSF1R, colony stimulating factor 1 receptor; FLA, fludarabine and cytarabine; FLT3, FMS-like tyrosine kinase 3; Fms, Feline McDonough sarcoma; GO, gemtuzumab ozogamicin; HD, Hodgkin disease; HD Ara-C, high-dose cytarabine; *HOX*, homeobox; HSCT, hematopoietic stem cell transplantation; IDH2, isocitrate dehydrogenase 2; MDS, myelodysplastic syndrome; JMML, juvenile myelomonocytic leukemia; KAT6A, K(lysine) acetyltransferase 6A; KMT2A, histone-lysine N-methyltransferase 2A; MAC, myeloablative conditioning; MerTK, MER proto-oncogene tyrosine kinase; MF, myelofibrosis; MM, multiple myeloma; MPAL, mixed-phenotype acute leukemia; MPN, myeloproliferative neoplasm; NHL, non-Hodgkin lymphoma; NK, natural killer cell; NPM1, nucleophosmin 1 gene; NUP214, nucleoporin 214 gene; NUP98, nucleoporin 98 gene; Ph, phase; PICALM, phosphatidylinositol binding clathrin assembly protein; r, rearrangement; R/R, relapsed or refractory; RIC, reduced intensity conditioning; RT, Richter transformation; TBI, total body irradiation; TMLI, total marrow and lymphoid irradiation; T-PLL, T-cell-prolymphocytic leukemia; and UBTF-ITD, upstream binding transcription factor-internal tandem duplication.

## Data Availability

No new data were created or analyzed in this study. Data sharing is not applicable to this article.

## Abbreviations

ALL	Acute lymphoblastic leukemia
ALAL	Acute leukemia of ambiguous lineage
AML	Acute myeloid leukemia
ASXL1	Additional sex combs-like 1
BCL-2	B-cell lymphoma 2
BP	Blast phase
CAR T-cell	Chimeric antigen receptor T-cell
CBF	Core-binding factor
CBF-AML	Core-binding factor acute myeloid leukemia
CD	Cluster of differentiation
CK1a	Casein kinase 1 alpha
CLL	Chronic lymphocytic leukemia
CML	Chronic myeloid leukemia
CMML	Chronic myelomonocytic leukemia
COG	Children’s Oncology Group
CR	Complete remission
CREBBP	CREB-binding protein
CSF1R	Colony-stimulating factor 1 receptor
DNMT3A	DNA (cytosine-5)-methyltransferase 3 alpha
DOT1L	Disruptor of telomeric silencing 1-like
DS-AMKL	Down syndrome-associated acute megakaryoblastic leukemia
EFS	Event-free survival
ELN	European LeukemiaNet
EP300	E1A binding protein p300
EZH2	Enhancer of zeste homolog 2
FDA	Food and Drug Administration
FLAG-IDA	Fludarabine, cytarabine, granulocyte colony-stimulating factor, idarubicin
FLT3	FMS-like tyrosine kinase 3
FLT3-ITD	FMS-like tyrosine kinase 3 internal tandem duplication
Fms	Feline McDonough sarcoma
FOLR1	Folate receptor alpha
GLIS2	GLIS family zinc finger 2
GO	Gemtuzumab ozogamicin
HD	Hodgkin disease
HDAC	Histone deacetylase
HD Ara-C	High-dose cytarabine
HMA	Hypomethylating agent
HOX	Homeobox
HRAS	Harvey rat sarcoma viral oncogene homolog
HSCT	Hematopoietic stem cell transplantation
IDH1	Isocitrate dehydrogenase 1
IDH2	Isocitrate dehydrogenase 2
JAK	Janus kinase
JMML	Juvenile myelomonocytic leukemia
KIT	KIT proto-oncogene receptor tyrosine kinase
KMT2A	Lysine methyltransferase 2A
KMT2A-r	KMT2A rearranged
KRAS	Kirsten rat sarcoma viral oncogene homolog
LSD1	Lysine-specific demethylase 1
MAC	Myeloablative conditioning
MAPK	Mitogen-activated protein kinase
MDS	Myelodysplastic syndrome
MECOM	MDS1 and EVI1 complex locus
MEIS	Meis homeobox
MF	Myelofibrosis
MLL	Mixed lineage leukemia
MNX1	Motor neuron and pancreas homeobox 1
MPAL	Mixed-phenotype acute leukemia
MPN	Myeloproliferative neoplasm
MRD	Minimal residual disease
MYST	MOZ, Ybf2/Sas3, Sas2, Tip60 family
NHL	Non-Hodgkin lymphoma
NK	Natural killer cell
NPM1	Nucleophosmin 1
NRAS	Neuroblastoma RAS viral oncogene homolog
NUP98	Nucleoporin 98
NUP214	Nucleoporin 214
OS	Overall survival
Ph	Phase
PI3K	Phosphoinositide 3-kinase
RAS	Rat sarcoma viral oncogene
RACE	Research to Accelerate Cures and Equity
R/R	Relapsed or refractory
RIC	Reduced-intensity conditioning
RT	Richter transformation
SAM	Sterile alpha motif
TBI	Total-body irradiation
TET2	Ten-eleven translocation 2
TMLI	Total marrow and lymphoid irradiation
T-PLL	T-cell prolymphocytic leukemia
UBTF-ITD	Upstream binding transcription factor internal tandem duplication
